# Chloroplast genome and phylogenetic analyses of *Poncirus trifoliata* (Rutaceae)

**DOI:** 10.1080/23802359.2019.1687023

**Published:** 2020-06-01

**Authors:** Shui-Lian He, Yang Tian, Yang Yang, Chong-Ying Shi

**Affiliations:** a College of Horticulture and Landscape, Yunnan Agricultural University, Kunming, China; b Yunnan Key Laboratory of Biomass Big Data, Yunnan Agricultural University, Kunming, China; c College of Science, Yunnan Agricultural University, Kunming, China; d Institute of Food Science and Technology, Yunnan Agricultural University, Yunnan, China

**Keywords:** Poncirus trifoliata, medicinal plant, chloroplast genome, phylogenetic analysis

## Abstract

*Poncirus trifoliata* is an important medicinal plant that is used to treat human diseases. In this study, the complete chloroplast (cp) genome of *P. trifoliata* was assembled based on the Illumina sequencing reads. The cp genome of *P. trifoliata* was 160,260 bp and contained two short inverted repeat regions (27,029 bp) which were separated by a small single copy region (18,760 bp) and a large single copy region (87,442 bp). The cp genome encodes 113 unique genes, including 79 protein-coding genes, 30 transfer RNA genes and 4 ribosomal RNA genes. The topology of the phylogenetic tree showed that *P. trifoliata* is closely clustered with genus *Citrus*.

*Poncirus trifoliata* (L.) Raf. (Rutaceae) known as trifoliate orange or Korean bitter orange is a deciduous or semi-deciduous shrub, native to China and Korea. Traditionally, *P. trifoliata* has been widely used in folk medicine as a remedy for gastritis, dysentery, inflammation and digestive ulcers. *P. trifoliata* fruit derived compounds have been reported to have various biological activities including anti-inflammatory, antibacterial and antianaphylactic (Kim et al. 1999), apoptosis of cancer cells (Rahman et al. 2015) and antilisterial (Rahman et al. 2012) properties. In order to clarify the taxonomical position of *P. trifoliata* in Rutaceae, We applied the Illumina technology to sequence, assemble and annotate the whole chloroplast genome of *P. trifoliata*. The resultant data have been made publicly available as a resource for genetic information for *Poncirus* species, and will provide a valuable plastid genomic resource for the future genetic and phylogenetic studies about *P. trifoliata.*

The fresh leaves of *P. trifoliata* were collected from the field of Kunming (25.20°N, 102.86°E). The voucher specimen was deposited at Herbarium of Yunnan Agricultural University (No. 2019HSL005). Total genomic DNA was isolated from fresh leaves using a DNeasy Plant Mini Kit (QIAGEN, Valencia, California, USA) according to the manufacturer’s instructions to construction chloroplast DNA libraries. The Illumina sequencing was conducted by Biomarker Technologies Inc. (Beijing, China). Resultant clean reads were assembled using GetOrganelle pipeline (https://github.com/Kinggerm/GetOrganelle). The genome was automatically annotated by using the CpGAVAS pipeline (Liu et al. 2012) and start/stop codons and intron/exon boundaries were adjusted in Geneious R11.0.2 (Biomatters Ltd., Auckland, New Zealand). All the contigs were checked against the reference genome of *Citrus aurantiifolia* (NC024929).

The complete chloroplast genome of *P. trifoliata* was 160,260 bp in length (Genbank accession number: MN102360). It was the typical quadripartite structure and contained contained two short inverted repeat (IRa and IRb) regions (27,029 bp bp) which were separated by a small single copy (SSC) region (18,760 bp) and a large single copy (LSC) region (87,442 bp). The cp genome encodes 113 unique genes, including 79 protein-coding genes, 30 transfer RNA (tRNA) genes and 4 ribosomal RNA (rRNA) genes. Twenty-four gene species are partially or completely duplicated, including twelve PCG (*ndhB*; *ndhF*; *orf56*, *rpl2*; *rpsl23*; *rps19*; *rps12*; *rps7*; *ycf1*; *ycf2; ycf15; ycf68*), seven tRNA (*trnI-GAU*, *trnA-UGC*, *trnL-CAA*, *trnI-CAU*, *trnR-ACG*, *trnV-GAC*, *trnN-GUU*), all four rRNA (4.5S, 5S, 16S & 23S rRNA) and one pseudogene (Ψ*rpl22*). The overall GC content of the cp genome was 38.4%, while that of LSC, SSC and IR regions was 36.8%, 33.3% and 42.9%, respectively.

A total of 17 cp genome sequences were selected to infer the phylogenetic relationships among the main representative species of Rutaceae with *Magnolia yunnanensis* (NC024545, Magnoliaceae) as outgroup. The combined datasets based on plastid genomes were aligned by MAFFT v7.307 (Katoh and Standley 2013). A neighbour-joining (NJ) phylogenetic tree was constructed in Geneious 11.1.5 (Kearse et al. 2012) with the Tamura-Nei genetic distance model, and a total of 1000 bootstrap replicates were performed. The topology of the phylogenetic tree showed that *P. trifoliata* has a close relationship with genus *Citrus* (Figure 1). The complete cp genome information reported in this study will be a valuable resource for future studies on genetic diversity, taxonomy and phylogeny of the Rutaceae.

**Figure 1.**
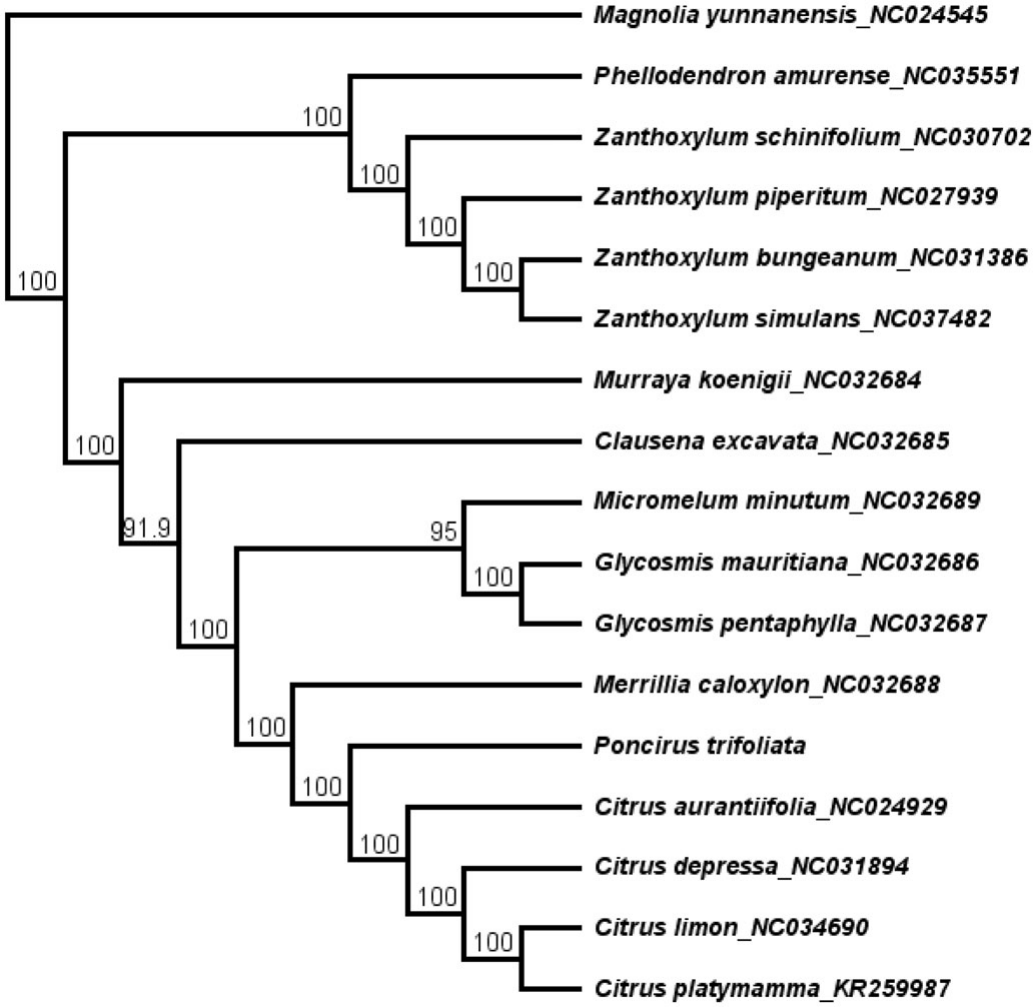
The neighbor-joining (NJ) phylogenetic tree based on 11 complete chloroplast genome sequence. Numbers at the right of nodes are bootstrap support values.
